# Consequences of Paternal Nutrition on Offspring Health and Disease

**DOI:** 10.3390/nu13082818

**Published:** 2021-08-17

**Authors:** Pauline Dimofski, David Meyre, Natacha Dreumont, Brigitte Leininger-Muller

**Affiliations:** 1 Nutrition-Génétique et Exposition aux Risques Environnementaux (NGERE), Institut National de la Santé et de la Recherche Médicale (Inserm), Université de Lorraine, F-54000 Nancy, France; pauline.dimofski@univ-lorraine.fr (P.D.); david.meyre@univ-lorraine.fr (D.M.); natacha.dreumont@univ-lorraine.fr (N.D.); 2 Service de Biologie-Biologie Moléculaire-Nutrition, CHRU-Nancy, Université de Lorraine, F-54000 Nancy, France

**Keywords:** paternal nutrition, metabolic outcomes, pregnancy health, epigenetic

## Abstract

It is well established that the maternal diet during the periconceptional period affects the progeny’s health. A growing body of evidence suggests that the paternal diet also influences disease onset in offspring. For many years, sperm was considered only to contribute half of the progeny’s genome. It now appears that it also plays a crucial role in health and disease in offspring’s adult life. The nutritional status and environmental exposure of fathers during their childhood and/or the periconceptional period have significant transgenerational consequences. This review aims to describe the effects of various human and rodent paternal feeding patterns on progeny’s metabolism and health, including fasting or intermittent fasting, low-protein and folic acid deficient food, and overnutrition in high-fat and high-sugar diets. The impact on pregnancy outcome, metabolic pathways, and chronic disease onset will be described. The biological and epigenetic mechanisms underlying the transmission from fathers to their progeny will be discussed. All these data provide evidence of the impact of paternal nutrition on progeny health which could lead to preventive diet recommendations for future fathers.

## 1. Introduction

**Figure 1:**
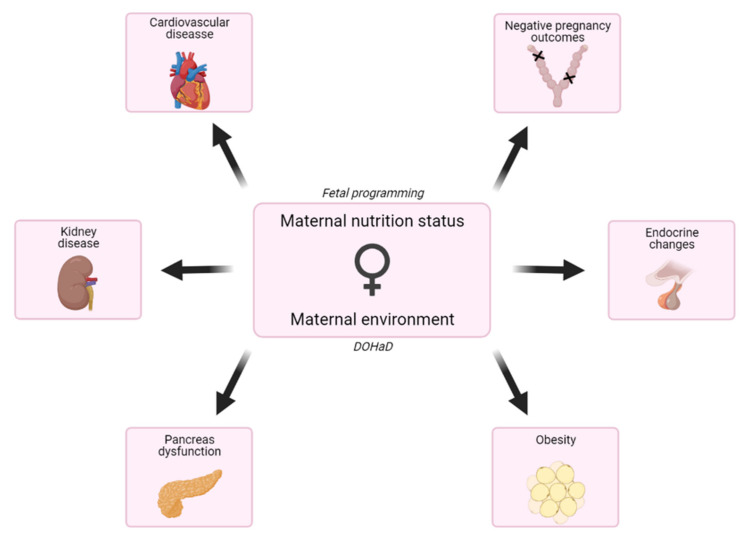
Effects of maternal nutrition status and environment exposition on the progeny. The fetal programming hypothesis postulates that changes occurring during fetal growth, such as a lack of nutrients, can predispose them to disease in adulthood. The fetal programming hypothesis is completed with the Developmental Origins of Health and Disease (DOHaD) concept, which includes exposition to toxins and chemicals, use of drugs, infections and other stimulations but also nutritional status during pregnancy, and early life.

It is now well established that maternal imbalanced nutrition during in utero period impacts offspring’s health and development. The fetal programming (the Barker) hypothesis (or DOHaD, developmental origins of health and diseases) stems from the link between maternal exposure to environmental factors and the development of complex diseases in adulthood. It postulates that changes occurring during fetal growth, such as a lack of nutrients or any environmental stress, can predispose to developing complex diseases in adulthood [1,2] (Figure 1). 

While the consequences of maternal diet on offspring health have been studied for 40 years, they remain partially understood (for review, see [3]). Since the 80s, it has been recognized that maternal folate deficiency is associated with neural tube defects (NTD) and intra-uterine growth retardation in the offspring [4]. These observations led to the World Health Organization (WHO) current recommendations, according to which all women should take a daily folic acid supplement from the pre-conceptional period until 12 weeks of gestation to prevent NTD [4]. Alteration of the maternal folate status also has consequences on birth weight, glucose blood levels, and insulin resistance (reviewed in [5]). Our previous work using a unique, well-defined animal model based on rats born from dams fed a methyl-deficient diet during gestation and lactation reported neurosteroidogenesis and epigenetic alterations associated with cognitive and motor disorders during offspring postnatal cerebral development [6,7,8]. Many studies show that maternal micronutrient deficiency is associated with an increased risk of cardiovascular disease, obesity, and type 2 diabetes in the offspring. Macronutrients like proteins or fat are also concerned, as suggested by several studies showing that a low or a high protein intake during pregnancy is linked to adverse health outcomes for the offspring [9]. Similarly, the quality but not the quantity of fat and adequate supply of vitamins and minerals plays a critical role during fetal development [10,11,12].

A growing body of evidence now suggests that the male environment and feeding patterns may also impact the progeny. To complete the DOHaD theory, the concept of Paternal Origins of Health and Disease (POHaD) was recently introduced (for review, see [13]). Reports have shown a decline in sperm quality in the past decades, likely due to environmental expositions or lifestyle factors [14]. Reproductive disorders are numerous and differ between men and women, the most common being infertility [15]. One of the common factors leading to infertility is the environmental exposure to toxins before conception, which influences sperm epigenetic processes (for review, see [16]). These toxics include environmental pollutants [17], endocrine-disrupting chemicals [18,19], and pharmaceutical drugs [20]. Lifestyle factors such as diet and exercise [21], alcohol and tobacco consumption [22], obesity, and trauma and stress situations [16] can influence fertility rates in humans. Recent studies suggest that the father’s environment may also impact fetal development and adult offspring health (for review, see [23]), considering the exposition to toxins or an altered diet could have epigenetic transgenerational consequences [19,24,25,26]. Deleterious effects issued from fathers are transmitted to the next generations via sperm. The underlying mechanisms are still poorly understood, but there is a growing body of evidence that small non-coding RNAs (sncRNAs) present in the sperm also play a pivotal role [27,28,29]. According to Dupont et al. (2019), transmission modalities are different between mothers and fathers. Transgenerational epigenetic inheritance from an exposed gestating female occurs when the transmission of the phenotypic alteration reaches the F3 generation, whereas it occurs in F2 when the male is exposed. Otherwise, the mode of inheritance is classified as intergenerational [27].

Most of the reported studies focused on rodent models. The epigenetic nature of transgenerational effects was initially mentioned by the pioneering Dutch famine [30,31] or the Overkalix cohort [32,33,34,35] studies. Many rodent models have been developed with various dietary modifications. The timing of animals’ exposure to these diets is critical and clearly influences the various outcomes described in these studies. This report focuses on the metabolic and molecular impacts of paternal diet (Table 1) on the offspring’s health. It mainly concerns the most described low nutrient diets like low-protein, fasting, or folic acid deficiency as well as high nutrient diets, like high sugar or high-fat diet which both are a health concern. The possible underlying molecular and epigenetic mechanisms will be discussed.

## 2. Epigenetic Mechanisms Involved in the Transgenerational Transmission

**Figure 2:**
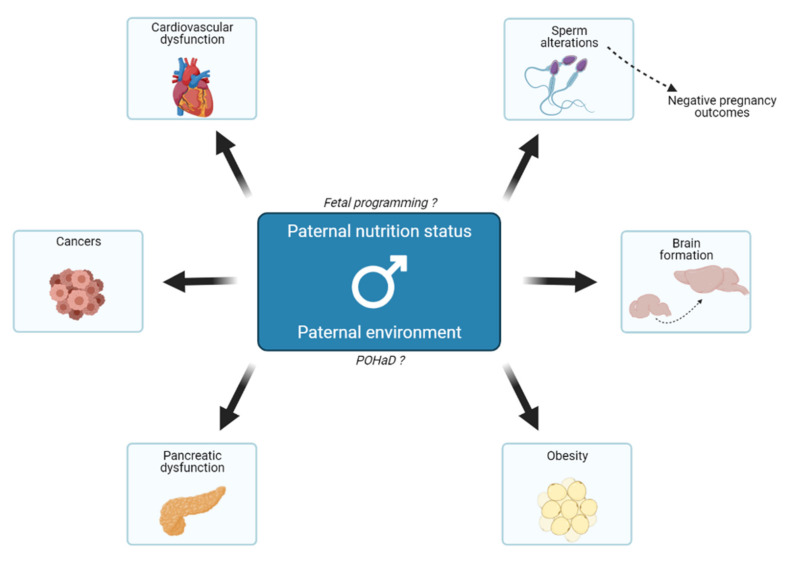
Effects of paternal nutrition status and environment on their offspring. The paternal nutritional status can act on offspring’s growth and metabolism and, therefore, its predisposition to various diseases in adulthood. That is related to the fetal programming hypothesis because fathers act indirectly on fetal growth. Besides, in the same way as the DOHaD theory, the POHaD paradigm suggests that the paternal environment could also influence the offspring’s metabolic changes. Mechanisms underlying all these events are poorly understood, but emerging evidence suggests that it could be transmitted through the sperm epigenome, notably in the case of negative pregnancy outcomes.

It is now evident that paternal exposure to environmental cues, especially an imbalanced diet, can affect the later development of the progeny (Figure 2). 

Effects are observed in the F1 generation (intergenerational transmission) and the subsequent ones (transgenerational transmission). This suggests that molecular clues are transmitted through the sperm and impact gene expression in the progeny. The likeliest scenario is that epigenetic marks are delivered by sperm to the zygote. With the advances in the field of epigenetics, the mechanisms responsible for this transgenerational inheritance begin to be better understood, with evidence pointing towards the involvement of DNA methylation, histone modifications, and above all, sncRNAs. All these events can interfere with each other [59] and are transmissible through the germline to the next generation without the intervention of genetic mechanisms (for reviews, see [28,60,61]; Table 2). The methylation process is allowed by methyl donors’ availability provided by one-carbon metabolism, which is an essential process for DNA methylation and DNA, RNA, and protein synthesis. Methionine is the precursor of SAM, the primary methyl group donor in the body’s methylation processes. The availability of methionine is dependent on the folate and methionine cycles. Therefore, folate status directly impacts epigenetic modifications [62]. Methylation reactions near gene promoter regions and histones can impact the transcriptional regulation and make the promoter regions inaccessible to transcription factors, and, therefore, influence the transcription of genes. For example, a father’s low protein diet in mice impairs tRNA fragments content in the sperm during its maturation, which are responsible for regulating gene expression [25,63]. Moreover, there are also presumptions about a father transmission to offspring involving small RNAs in mice [64,65,66], which are detailed in recent reviews [27,28,29].

DNA methylation is the most studied epigenetic mark. It is a dynamic modification that is erased and reset during two rounds of reprogramming, one in primordial germ cells during gametogenesis and the other one later in the preimplantation embryo. Mice studies on various nutritional models, including high fat, low protein, undernutrition, or folate deficiency, suggest that only a small number of sperm loci are differentially methylated according to paternal feeding [24,49,51,70,71,72]. Moreover, a limited subset of them is maintained in offspring’s tissues [73]. In a paternal mouse model fed a high-fat diet and leading to glucose intolerance in the F1 offspring, a genome-wide comparison between the transcriptome and DNA methylation indicates that differential DNA methylation is not responsible for changes in gene expression [74]. 

The role of histone modifications in paternal intergenerational inheritance is unclear, mainly because only a few studies have focused on these epigenetic marks. Moreover, chromatin in sperm displays a different organization than in somatic tissues. Histones are replaced by transition proteins and then by protamines during spermatogenesis. Only a small fraction of the human sperm chromatin escapes this remodeling [75]. The fact that the remaining sperm histone modifications are transmitted to the embryo suggests a role beyond fertilization. However, histone positioning was similar in sperm from lean or obese men, whereas DNA methylation and sncRNA content were modified [76]. Feeding male mice with a high-fat diet for ten weeks induced H3K4me1 enrichment at transcription regulatory genes compared to controls and changes in hepatic expression levels of 7 genes in the offspring [77]. Paternal sugar in Drosophila was reported to alter H3K9 and H3K27me3 deposition in the zygote, with chromatin state rewiring later in the adult progeny, which was associated with obesity susceptibility [78]. To further point towards a role of histone modification in transgenerational inheritance despite the lack of studies, mice paternal overexpression of the gene coding for the H3K4 demethylase (KDM1A, also known as LSD1) affects development and survival in offspring [79].

Last but not least, sncRNA content in the sperm is affected by diet and has been the center of interest in recent years. Despite cytoplasm and RNA are ejected at the end of spermatogenesis, there remain mRNA fragments and several types of sncRNA in mature spermatozoa: the well-described miRNAs, piRNAs, endo-siRNAs, and tRNA-derived fragments, these being the most abundant in the spermatozoa [29]. Recent studies suggest that these sncRNAs are the molecules enabling the paternal transmission to the progeny, as injecting sperm RNA content in fertilized oocytes is sufficient to recapitulate the phenotype in the progeny. Effects of a high-fat or low protein diet on sncRNA were the most described (reviewed in [28]), with a significant role of tRNA-derived fragments. tRF-Gly-GCC levels were increased in the sperm of male mice fed a low protein diet. The expression of targets of these tRNA-derived fragments was consequently diminished in embryos [25].

## 3. Impact of Paternal Folic Acid Intake on Progeny’s Health

### 3.1. Paternal Folate Deficiency

Folate, an essential vitamin B, is a pivotal molecule in the one-carbon metabolism. Folate participates in S-adenosylmethionine (SAM) synthesis, which plays a significant role in many biological processes like DNA, RNA, and protein methylation [80]. SAM is also essential in the amino acid [80] or lipid metabolism and hormonal synthesis [81] or nucleotide synthesis [64]. Since the 80s, it is recognized that maternal folate deficiency is associated with NTD and more subtle changes in offspring development [4]. A lowered maternal folate status has other consequences in offspring, such as intrauterine growth retardation, low birth weight, hyperglycemia, and insulin resistance (reviewed in [5]). There are emerging shreds of evidence that paternal folate intake also impacts the offspring’s health and disease onset. A recent study highlighted that offspring of paternal folate deficient rats are more susceptible to develop anxious and depressive traits [36]. In 2013, a mouse study reported by Lambrot et al. showed that a low paternal folate intake during the periconceptional period alters the sperm epigenome and can be linked to adverse pregnancy outcomes, such as a reduced pregnancy rate post-implantation loss, fused and abnormal placentas [49]. Folate status influences reproductive health in humans, especially the gestation duration when fathers have a 4 mg/day folate intake [52] (for review, see [82]). There is a higher frequency of developmental abnormalities in mice like craniofacial malformations, limb defects, muscle and/or skeletal development delay [49]. Moreover, offspring of the folate-deficient fathers (0.3 mg/kg) display lateness in the meiosis appearance at postnatal day 12. No significant difference in sperm counts, spermatogenesis, nor testis morphology was observed in adulthood [49], while Yuan et al. found an impaired spermatogenesis process in the same conditions [56], and Swayne et al., report a decreased sperm count [57]. Other rodent studies showed paternal folate deficiency impacts placental folate transport [53], DNA methylation and/or mutation [57,58,83,84], and Igf-2 expression level in the fetal brain [83,84].

### 3.2. Paternal Folate Supplementation

Paternal supplementation is also being investigated. According to Ly et al., it might affect embryo abnormalities with an increase in the offspring sired by F1 males fed with a 20-fold folate sufficient diet compared with folate-deficient fathers [51]. The authors found that there was no difference in the number of pups or the body weight in the F1 litter sizes at birth, but they measured a significant decrease in the sperm count at postnatal day 200 when males were fed 20-fold folate sufficient diet (40 mg/kg) [51]. At weaning, a supplemented diet negatively impacted the F2 litter sizes and increased the postnatal mortality in the same manner as when fathers were fed with a folate-deficient diet [51]. Furthermore, elevated paternal methyl-donor consumption affects cognitive and neural function, especially hippocampus-dependent learning and memory function and hippocampal synaptic plasticity [85]. Chicken studies highlighted that paternal folic acid supplementation could impact spermatozoa mRNA expression [44] and induce transgenerational metabolic changes [68].

In summary, it appears that both folate deficient and supplemented diets could have an intergenerational impact on the offspring [86]. Thus, paternal folate intake is as crucial as maternal intake, and it must be taken into consideration for further recommendations.

## 4. Depleted Paternal Diets and Their Effects on the Progeny

### 4.1. Impact of a Paternal Low-Protein Diet 

Obesity results from an imbalance between energy intake and energy expenditure and is influenced by genetic predisposition and/or epigenetic inheritance and transgenerational effects [87,88]. For instance, the protein content of the paternal diet influences offspring metabolism. When fathers are fed a low protein diet (LPD) in mice, pups are more prone to glucose intolerance, metabolic and cardiovascular dysfunctions, impaired skeletal development, and altered patterns of bone mineral deposition [24,37,38,50]. Moreover, male pups of LPD fathers are heavier [37], whereas female pups are lighter than control pups [54]. Alteration in offspring vascular function was also reported [89] and an increased risk of developing breast cancer in female offspring [54]. Offspring gene expression is altered, especially genes involved in cholesterol and lipid synthesis pathways [24,90]. It also negatively affects gene expression of various AMPK pathways in the blastocysts but positively affects genes regulating fetal growth, with up-regulation of genes coding for nutrient transporter in the placenta and imprinted genes involved in fetal growth regulation [38,54]. Taken together, these results suggest that paternal LPD could have an adverse effect on the offspring, with females slightly more affected than males.

### 4.2. What about Paternal Food Restrictions?

Since the Dutch famine, there are pieces of evidence that poor maternal nutrition during the conception period could lead to negative consequences, not only for mental but also for physical health. Most studies focused on later life health, considering the exposure time, and developing tissues or organs, the early gestational period being the most vulnerable time. Offspring of people subjected to famine has an increased risk of type-2 diabetes, heart disease, depression, and schizophrenia, as well as premature aging [86]. Similar effects seem to occur in undernourished African populations (for review, see [86]). However, most of these data concern mother exposition to undernutrition, and there are few studies concerning father fasting. When fathers are fasting, both male and female progeny display a decreased glycemia and an increased serum corticosterone level, but there are also changes in the serum IGF-1 concentration in the offspring [91]. Using a 70% restriction diet, McPherson et al. reported that paternal food restriction impairs preimplantation embryo development, global methylation, and function of sperm with possible consequences on their fertility. This caloric restriction also had consequences of a reduced postnatal weight and growth retardation, with an increase of dyslipidemia and adiposity prevalence in the offspring [21]. A study in *Caenorhabditis elegans* also highlighted that paternal food restriction affects both males’ and females’ progeny’s fat content [92]. McPherson et al. further investigated the father’s antioxidant and vitamin supplementation (vitamin B9, C, E, lycopene, selenium, zinc, and green tea extract) and showed that oxidative status in their sperm is correlated with the progeny growth [21]. Supplementation of food-restricted fathers reversed the effects on offspring’s adiposity and weight. Food restriction affected gene expression in a sex-specific manner in the progeny and was partially reversed by antioxidant and vitamin supplementation [21]. Human [93] and rodents [94,95] studies provide evidence that food restriction also positively impacts blood glucose levels, insulin sensitivity, adiposity, and type-2 diabetes risk. As for the effects at the cellular level, many studies underline the involvement of DNA repair, autophagy, adaptive cellular stress response signaling pathways, mitochondrial health, stem cell-based regeneration, and long-term metabolic effects in the mechanisms related to intermittent fasting [96]. Another study developed on mice embryos suggests that mothers could modulate the effects of paternal caloric restriction on offspring development. In fact, the authors observed absence or reversed effects on the progeny between embryo transfer and natural mating [97]. Furthermore, paternal dietary changes before conception could impact offspring’s behavior, since an anxiolytic-like behavior has been described in adulthood [98].

## 5. Effects of Enriched Paternal Diets on Offspring

### 5.1. Consequences of a Paternal High-Fat Diet 

A paternal high-fat diet (HFD) may influence metabolic outcomes and obesity in human offspring [99,100] (for review, see [101]). In animal models, this diet is known to induce a transgenerational body-weight reduction in the rat pups at postnatal day 3 [39], but an increase for older offspring in mice [40,48,102,103] with a higher body fat mass [41,102,103]. Rodent fathers fed with an HFD can transmit pancreatic β-cell dysfunction and glucose intolerance to their F1 female offspring [45] and an altered response to insulin in F1 male offspring [40]. Further investigations on retroperitoneal white adipose tissue demonstrate that numerous metabolic pathways in offspring are impacted, such as cellular and mitochondrial response to stress, cell survival and death, telomerase signalling, cellular growth and proliferation, and cell cycle [104]. Paternal consumption of HFD gives genetic signatures linked to chronic degenerative diseases and early aging, both in pancreatic β-cell and retroperitoneal white adipose tissue of their offspring [45,104]. Furthermore, paternal HFD exposure can lead to an increased risk of chronic kidney disease [102] and to the development of a metabolic syndrome-like phenomenon [103]. Moreover, Zhou et al. report that paternal HFD results in cognitive impairments in the F1 pups but not in the F2 generation in mice. These impairments are linked to decreased hippocampal neurogenesis. These results are potentially due to the increased methylation of the *BDNF* gene promoter transmitted by F0 spermatozoa [105]. When fathers are fed an HFD, mating female mice show a reduced number of plugs and pregnancies, possibly because of their 20% sperm motility decreases [41]. In vitro fertilization with sperm from fathers fed with an HFD shows a delay in the preimplantation development of embryos and a reduced frequency of blastocyst formation, and a decreased ability of embryos to implant in the uterine wall. Blastocyst biological processes are also concerned; changes in the carbohydrate metabolism with an increased glycolysis pathway and pyruvate uptake, and reduced mitochondrial membrane potential [42,48]. Additional reports showed that it could also lead to adverse pregnancy outcomes like a reduced rate of ongoing pregnancy, placental and fetal weights, and morphological retardation such as a retarded limb morphology and a decreased crown–rump length [42,48]. These data suggest that paternal HFD negatively impacts embryo physiology and its adult health status in rodents.

### 5.2. Impacts of a Paternal High-Sugar Diet 

As a low-protein diet or an HFD, paternal high sugar nutrition adversely affects their offspring. A high-sugar diet is a health concern. It is known that this diet has a deleterious impact on human physiology [43,55]. Excess intake of sugars like glucose or fructose has consequences on cardiovascular and metabolic diseases. Fructose is currently added to industrialized food [106], and its overconsumption induces an increased hepatic triacylglycerol concentration, hypercholesterolemia, hypertriglyceridemia, and an alteration in glucose metabolism with hyperinsulinemia in both mothers and fathers [46,47]. Consequently, for their offspring, if mother or father alone, or both, are subjected to a high-fructose diet, a decreased adiponectin concentration but an increased leptin level, as well as increased blood pressure, uricemia, and genital fat are observed [46,47]. When parents are both fed a high-fructose diet, there are more severe effects on offspring’s liver metabolism in association with increased inflammatory markers [46] (for review, see [107]). In the same way, another study in *Drosophila* shows that a paternal high-sugar diet could be linked to altered energy homeostasis in the offspring. However, it did not affect the growth and the development of the offspring, but stably reprogramed their metabolism [67,78,108]. The study conducted by Nätt et al. show that RNA sperm contents change rapidly in response to a high-sugar diet in human. They used a two-step strategy in which each participant was his own control [69]. In this study, they reported that the sperm count was unaffected, but some specific subtypes of tsRNAs were up-regulated in response to a high-sugar diet. Then, they compared with obese men who also have an alteration in their tsRNAs sperm contents [69]. The combination of high-sugar with a high-fat diet results in a dysfunction of the reproductive system with an earlier testicular descent in the F1 generation [109]. The authors also observed a statistical difference in the A and B phases penile glans morphology ages in F1 and F2 generations when males are fed a hypercaloric diet. This hypercaloric diet is also known to increase fat mass in rats [80,109] and in mice [64,81]. These earlier studies suggest that a high sugar or a hypercaloric diet could affect both the offspring’s metabolism and reproductive parameters.

## 6. Conclusions and Perspectives

The maternal nutrition status strongly influences fetal development and growth, but it becomes more and more evident that both parents’ nutritional status acts on offspring’s health and disease in adulthood (for review, see [110]). Diet modifications in fathers, whether it is an excess or a restriction of nutrients, lead to negative pregnancy outcomes and/or metabolic changes in their progeny. Changes during critical periods of the embryo and fetal development may have long-term consequences [59,111]. The differences between the duration and/or the period of exposition to an altered diet have to be considered, as well as the model that is used, animals or a human study. For instance, a paternal HFD exposition before mating induces a decreased [39] body weight during early postnatal days in the mice offspring while it is increased at later life in a rat model [40,48]. Furthermore, another study made on rats shows that maternal, but not paternal, HFD affects both F1 male and female offspring’s phenotypic parameters (blood glucose level, plasma insulin, and leptin, HOMA, percentage of body fat). Plasma leptin levels are also affected in the F2 generation from F1 females fed an HFD [112]. These results are inconsistent with the study of Masuyama et al. [103], who reported an effect of paternal diet on pup’s metabolism. An explanation could be the animal model used, rat vs. mice, and/or the differences between the diet composition used: 34% vs. 62% fat, 23% vs. 18% protein, 43% vs. 20% carbohydrate [103,112].

It is important to note that some inherited epigenetic signatures are preserved between species like the fly, mouse, and human [78]. Besides epigenetic mechanisms, human studies are focusing on the genetic aspects of POHaD. Kong et al. sequenced the entire genome of Icelandic people to study their genome-wide de novo mutation rate. The single-nucleotide polymorphism mutation rate diversity was linked to the father’s age at conception, with no effect of the mother’s age [113]. They further studied how sex-dependent differences in the maintenance and development of germ cells affect their mutability. They observed that the mother’s age was more responsible for the increased number of clustered mutations than the father’s age. The authors also highlighted that de novo mutation clusters are greater in the maternal genome than in paternal ones [114,115]. Both maternal and paternal age at conception are essential factors of transmitting de novo mutations to the progeny. Besides age, environmental exposures, including diets, may also impact de novo mutation rate in fathers with possibly transgenerational consequences. Further investigations are needed to understand how father’s diet and/or exposition to environmental factors can influence the disease onset of their children during adulthood. The literature on the topic in humans is slightly limited and should be prioritized in the future.

All these shreds of evidence highlight the importance of the father’s nutritional status during the periconceptional period for providing an efficient fetal development and growth of the progeny. All these events are adaptive and may change during life to confer, or not, predisposition for chronic disease onset in adulthood. Despite all these data, the underlying mechanisms remain poorly understood and should be investigated in the future in order to provide fathers with optimal dietary recommendations.

## Figures and Tables

**Table 1:** Paternal nutrition status effects.

Effects		Paternal Diet	References
Metabolism	Body weight and adiposity	Low protein HFD Food restriction FA deficient High sugar	[21,36,37,38,39,40,41,42,43]
	Glucose intolerance or hyperglycemia	Low protein HFD FA supplemented High sugar	[37,38,40,44,45,46,47]
	Insulin resistance or hyperinsulinemia	HFD High sugar	[40,45,46,47]
	Carbohydrate metabolism	HFD	[42,48]
	Lipid metabolism	High sugar FA deficient or supplemented Food restriction	[21,44,46,47]
	Uricemia	High sugar	[43,46,47]
Development	Skeleton	Low protein FA deficient	[49,50]
	Fetal or post-natal growth	Food restriction	[21]
	Spermatogenesis	FA deficient	[49]
	Embryo or placenta abnormalities	FA supplemented HFD	[42,48,51,52,53]
Pregnancy outcomes	Number of plugs/pregnancies	HFD FA deficient	[41,42,48,52]
	Pre or Post implantation development	HFD FA deficient Food restriction	[21,42,48,52]
	Morphological retardation	HFD FA deficient	[42,48,49]
	Postnatal mortality	FA deficient or supplemented	[51]
Organ and system dysfunction	Breast cancer	Low protein	[54]
	Diabetic kidney disease	High sugar	[55]
	Cardiovascular system	Low protein High sugar	[37,38,43,46,47,55]
	Sperm	HFD High sugar FA deficient or supplemented	[41,46,51,56,57]
	Endocrine system	High sugar	[46]
	Central nervous system	FA deficient	[36,58]

**Table 2:** Epigenetic and RNA alteration induced by fathers’ nutrition status.

Effects		Paternal Diet	References	Comments
Epigenetic alterations	Sperm DMRs ^1^	Low protein FA deficient or supplemented	[51,54]	Paternally imprinted gene H19
	DNA methylation	Low protein HFD FA deficient Food restriction	[38,39,49,56]	Genes involved in neurological and autoimmune disease, diabetes, cancer, autism and schizophrenia
	Gene expression and transcriptome	High sugar	[63,67]	H3K9-me3 regulators and Polycomb
	Sperm mutations	FA deficient or supplemented	[57]	DNA fragmentation and tandem repetitions
RNA changes	Sperm mRNA content and/or expression levels	Low protein HFD High sugar FA sufficient	[24,25,39,40,64,65,67,68,69]	Metabolic disorders, lipid metabolism, embryonic development and spermatogenesis
	sncRNA	Low protein High sugar	[25,69]	tRNA and tsRNA
		HFD FA supplemented	[39,68]	piRNA
		Low protein HFD FA supplemented	[25,39,40,44,64,65,66]	miRNA let-7 species miR-19 species miR-182 miR-183 miR-340 miR-193b miR-204

^1^ DMRs for differentially methylated regions.

## Data Availability

Not applicable.

## Abbreviations

DOHaD	developmental origins of health and diseases
HFD	high-fat diet
LPD	low protein diet
NTD	neural tube defect
PND	postnatal day
POHaD	paternal origins of health and disease
SAM	S-adenosyl methionine
sncRNAs	small non-coding RNAs
WHO	world health organization
